# New Views on Strand Asymmetry in Insect Mitochondrial Genomes

**DOI:** 10.1371/journal.pone.0012708

**Published:** 2010-09-15

**Authors:** Shu-Jun Wei, Min Shi, Xue-Xin Chen, Michael J. Sharkey, Cornelis van Achterberg, Gong-Yin Ye, Jun-Hua He

**Affiliations:** 1 State Key Laboratory of Rice Biology and Ministry of Agriculture Key Laboratory of Molecular Biology of Crop Pathogens and Insects, Institute of Insect Sciences, Zhejiang University, Hangzhou, China; 2 Institute of Plant and Environmental Protection, Beijing Academy of Agriculture and Forestry Sciences, Beijing, China; 3 Department of Entomology, University of Kentucky, Lexington, Kentucky, United States of America; 4 Department of Entomology, Nationaal Natuurhistorisch Museum, Leiden, The Netherlands; Natural History Museum of Denmark, Denmark

## Abstract

Strand asymmetry in nucleotide composition is a remarkable feature of animal mitochondrial genomes. Understanding the mutation processes that shape strand asymmetry is essential for comprehensive knowledge of genome evolution, demographical population history and accurate phylogenetic inference. Previous studies found that the relative contributions of different substitution types to strand asymmetry are associated with replication alone or both replication and transcription. However, the relative contributions of replication and transcription to strand asymmetry remain unclear. Here we conducted a broad survey of strand asymmetry across 120 insect mitochondrial genomes, with special reference to the correlation between the signs of skew values and replication orientation/gene direction. The results show that the sign of GC skew on entire mitochondrial genomes is reversed in all species of three distantly related families of insects, Philopteridae (Phthiraptera), Aleyrodidae (Hemiptera) and Braconidae (Hymenoptera); the replication-related elements in the A+T-rich regions of these species are inverted, confirming that reversal of strand asymmetry (GC skew) was caused by inversion of replication origin; and finally, the sign of GC skew value is associated with replication orientation but not with gene direction, while that of AT skew value varies with gene direction, replication and codon positions used in analyses. These findings show that deaminations during replication and other mutations contribute more than selection on amino acid sequences to strand compositions of G and C, and that the replication process has a stronger affect on A and T content than does transcription. Our results may contribute to genome-wide studies of replication and transcription mechanisms.

## Introduction

Most animal mitochondrial genomes are about 16 Kb in size and contain 37 genes: 13 protein-coding genes, 22 transfer RNA genes (tRNA) and two ribosomal RNA genes (rRNA) [1]. Additionally, there is an A+T-rich region which contains essential regulatory elements for the initiation of transcription and replication, and is therefore referred to the control region [2]. Features of highly economized organization, lack of recombination, maternal inheritance and a high mutation rate relative to the nuclear genome have resulted in the wide use of mitochondrial genomes in studies of genome evolution, population genetic structure and phylogenetic inference [3].

A remarkable feature of mitochondrial DNA is the violation of Chargaff's second parity rule, called strand asymmetry (strand compositional bias) [4], [5]. Strand asymmetry is usually reflected by AT skew, as expressed by (A−T)/(A+T), and GC skew, as expressed by (G−C)/(G+C) [6]. Positive AT skew values indicate more A than T on the target strand, and positive GC skew values indicate more G than C, and vice versa. In insect mitochondrial genomes, the two DNA strands are referred to as the majority strand (light strand in mammal mitochondrial genomes), on which more genes are coded, and the minority strand (heavy strand in mammal mitochondrial genomes) [7]. Additionally, there is usually more A than T and more C than G on the majority strand. However, in some arthropods [8], [9], [10], [11], [12], flatworms [13], brachiopods [14], echinoderms [15] and fish [16], strand asymmetry is reversed and there is less A than T and less C than G on the majority strand.

Replication and transcription processes, during which one strand is transiently in a single-stranded state and thereby exposed to more DNA damage, has been widely considered to bias the occurrence of mutations between the two complementary DNA strands [9], [17]. Therefore, inversion of the replication origin located in the A+T-rich region would change the replication order of two mitochondrial DNA strands and consequently lead to reversal of strand asymmetry [9]. It has been demonstrated in a crustacean [10] and in two vertebrates that replication order is responsible for the reversal of strand asymmetry [18]. Moreover, it has been demonstrated in experiments that rates of spontaneous deamination of A and C nucleotides are higher in single-stranded DNA than in double-stranded DNA [19], [20], [21]. Deamination of A yields a base, hypoxanthine, that pairs with C rather than T, while deamination of C yields a base, uracil, that pairs with A instead of G [22].

In order to decipher the underlying mutation processes causing strand asymmetry in mitochondrial genomes, much research has gone into assessing the contributions of different substitution types associated with replication. C deamination that promotes C:G to T:A transitions was shown to be the major source of mutation in vertebrate mitochondrial DNA [23], [24], [25]. Additionally, genes closer to the replication origin of leading strands remain exposed to mutation for a longer period of time during replication and this should result in a positive correlation between strand asymmetry and duration of time spent in the single-stranded state (DssH) [23]. However, different types of mutation were found to respond differently to DssH gradients [25]. There is no direct evidence to demonstrate the existence of transcription-associated mutational asymmetry in mitochondrial genomes although this phenomenon has been observed in enterobacteria [26], [27], [28] and in the nuclear DNA of eukaryotes [29], [30]. Another way to examine the mutation spectrum in mitochondrial DNA is to directly estimate the rates of different substitution types without discerning the processes of replication and transcription. In fruit fly and mouse mitochondrial DNA, transition of G:C to A:T is the dominating mutation [31], [32], but in a nematode A:T to G:C [33] is dominant, indicating that the spectrum of mitochondrial mutations varies across taxa [34]. In summary, the relative contributions of replication and transcription on strand asymmetry are still unclear.

Here, we conduct a broad survey of strand asymmetry in 120 insect mitochondrial genomes, with special reference to the correlation between skew values and gene direction/replication orientation.

## Results

### Reversal of strand asymmetry in insect mitochondrial genomes

**Figure 1:**
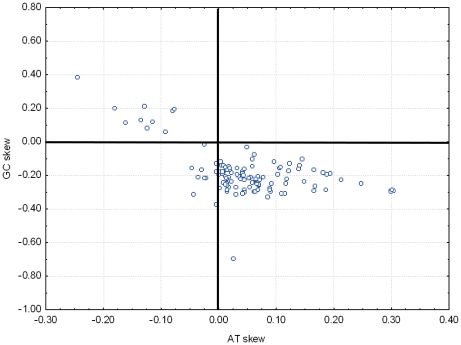
Scatterplots of skews values calculated for whole majority strand of 120 insect mitochondrial genomes. Reversal of both AT and GC skew are found in all species with a sequenced mitochondrial genome of in the families Philopteridae (*Bothriometopus macrocnemis, Campanulotes bidentatus*), Aleyrodidae (*Aleurochiton aceris*, *Aleurodicus dugesii*, *Bemisia tabaci*, *Neomaskellia andropogonis*, *Tetraleurodes acaciae*, *Trialeurodes vaporariorum*) and Braconidae (*Cotesia vestalis*, *Spathius agrili*). Reversal of AT skew was found in 10 unrelated species: *Onychiurus orientalis* (Collembola: Onychiuridae), *Bilobella aurantiaca* (Collembola: Neanuridae), *Heterodoxus macropus* (Phthiraptera: Boopidae), *Antheraea pernyi* (Lepidoptera: Saturniidae), *Coreana raphaelis* (Lepidoptera: Lycaenidae), *Manduca sexta* (Lepidoptera: Sphingidae), *Saturnia boisduvalii* (Lepidoptera: Saturniidae), *Simosyrphus grandicornis* (Diptera: Syrphidae), *Polystoechotes punctatus* (Neuroptera: Polystoechotidae) and *Abispa ephippium* (Hymenoptera: Eumenidae).

We calculated the AT and GC skews for the entire majority strand of 120 insect mitochondrial genomes. Most species have positive AT skews and negative GC skews, i.e., most have a strand asymmetry characterized by an excess of A relative to T and an excess of C relative to G. However, ten species, i.e., two species in Braconidae (Hymenoptera), two species in Philopteridae (Phthiraptera) and six species in Aleyrodidae (Hemiptera), showed negative AT skews and positive GC skews (Figure 1), implying that these species have strand asymmetry reversal on the entire majority strand, with less A than T and less C than G (Figure 1, Table S1). All species with sequenced mitochondrial genomes in the above three families showed reversal of strand asymmetry, suggesting that reversal of strand asymmetry may have occurred in basal members of these taxa and be phylogeneticly associated.

### A+T rich region and reversal of strand asymmetry

**Figure 2:**
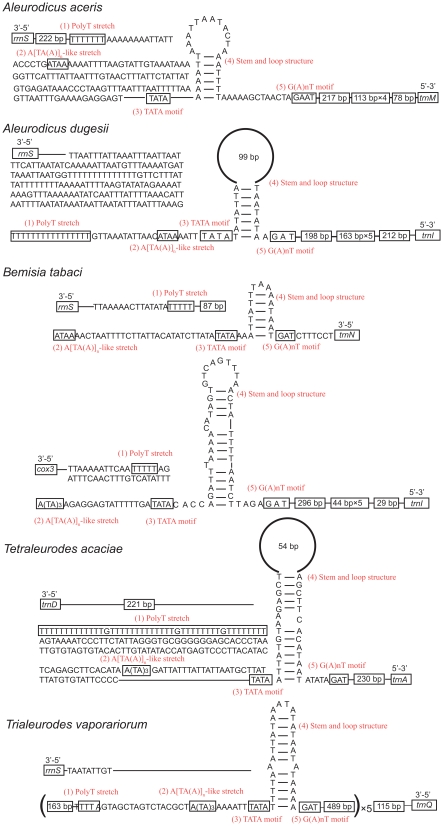
Predicted structural elements for A+T-rich region of 10 species with reversal of strand asymmetry.

Strand asymmetry is presumed to be predominantly due to the commonality of deamination of A and C in single-stranded DNA during replication and transcription, although the relative contributions of these two processes is uncertain [17], [35]. Based on replication theory, inversion of replication origin located in the A+T-rich region would lead to reversal of strand asymmetry [9], [10], [18]. However, it is not easy to detect an inversion of the A+T-rich region because this is the most variable region of the mitochondrial genome, making it impossible to align between distant species. Consequently, the orientation of the control region cannot be determined by simple sequence comparisons [12]. The direction of the A+T-rich region was determined in the 10 species with reversal of strand asymmetry on the entire majority strand by examination of the elements related to the regulation of transcription and control of DNA replication [2] (Figure 2, and Figure 2A and 2C in [36]).

For the family Braconidae, the A+T-rich region is located between *trnM* and *trnQ* both in *Cotesia vestalis* and *Spathius agrili*. Apart from the conserved elements in insect A+T-rich region, tandem repetition was also found to be a characteristic of A+T-rich regions [2]. In *S. agrili*, a repeat sequence is present at the 3′-end upstream of the *trnQ*. In both species, all elements related to the regulation of transcription and control of DNA replication in the A+T-rich region were present and arranged in the conserved order, except for the G+A-rich sequence downstream of the stem and loop structure, and an A[TA(A)]_n_-like stretch in *S. agrili*. However, all of these elements are in opposite directions and strands compared to those of other insects [2], [37], revealing an inversion of the A+T-rich region in these two species of Braconidae [10], [36].

Amongst Aleyrodidae, all species have one A+T-rich region except *Bemisia tabaci*, which has two, one small and one large. Tandem repeat sequences were found in the A+T-rich region of all species except for in the small one in *B. tabaci* [38]. All of these repeat regions are located at the 3′-end of the A+T-rich regions except for that of *Neomaskellia andropogonis*. Replication and transcription related elements in the A+T-rich regions are present in conserved order in all species except for *N. andropogonis* and *Tetraleurodes acaciae*. In *B. tabaci*, conserved elements were found in both A+T-rich regions. In *Trialeurodes vaporariorum* all conserved elements were found in each of five repeat sequences of the A+T-rich region, indicating that these elements were repeated five times. In *T. acaciae*, a long polyT stretch is present in the middle of the A+T-rich region upstream of the tandem repeat region. These elements are in opposite directions and strands as in two braconid species, indicating that the mitochondrial genomes of aleyrodid species also have an inversion of the A+T-rich region.

For the Philopteridae, there are several A+T-rich regions in each species and the inferred stem-loop structures in each A+T-rich region are located on the majority strand [8], [39], which might indicate reversals of the A+T-rich regions.

In summary, examination of regulatory elements in A+T-rich regions directly supports the hypothesis that the reversal of strand asymmetry is caused by inversion of replication origin.

### Gene arrangement and reversal of strand asymmetry

Mitochondrial gene arrangement is highly conserved in most animals [1], though there are exceptions. The 10 species with reversal of strand asymmetry over the entire mitochondrial genome were found to have accelerated gene rearrangement rates [8], [38], [40]. However, species that have accelerated gene rearrangement rates do not always show a reversal of strand asymmetry, e.g., three *Nasonia* species (Insecta: Hymenoptera) [41] and *Thrips imaginis* (Insecta: Thysanoptera) [42]. Below, we explore the relationship between gene arrangement and strand asymmetry in species with reversal of strand asymmetry over the entire mitochondrial majority strand following the traditional classification of rearrangement events: translocation, local inversion (inverted in the local position), shuffling and remote inversion (translocated and inverted) [43].

**Figure 3:**
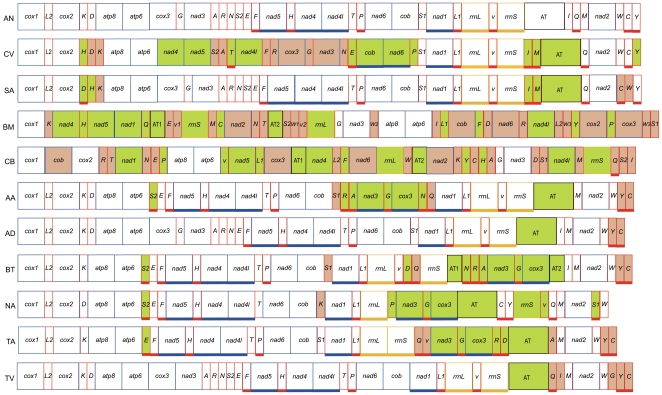
Gene arrangement of the mitochondrial genome of 10 species with reversal of strand asymmetry. Genes are showed in boxes. Box with underscore indicates that the gene is encoded on minority strand while box without underscore indicates that the gene is encoded on majority strand. Rearranged genes compared to the ancestral mitochondrial genome arrangement were marked with colours. Green indicates that the gene was inverted or remotely inverted, while brown indicates that the gene was translocated or shuffled. AT indicates the A+T-rich region. A+T-rich regions of all species were inverted and marked in green. AN: Ancestral arrangement; CV: *Cotesia vestalis*; SA: *Spathius agrili*; BM: *Bothriometopus macrocnemis*; CB: *Campanulotes bidentatus*; AA: *Aleurochiton aceris*; AD: *Aleurodicus dugesii*; BT: *Bemisia tabaci*; NA: *Neomaskellia andropogonis*; TA: *Tetraleurodes acacia*; TV: *Trialeurodes vaporariorum*.

For these 10 species, gene rearrangement varied greatly both among and within families (Figure 3). In Braconidae, the *S. agrili* mitochondrial genome is relatively conserved in gene arrangement with five tRNA genes rearranged. However, in *C. vestalis*, the mitochondrial genes are highly rearranged with 15 tRNA and seven protein-coding genes inverted, representing 5968 bp, more than one third of the genome [36]. As discussed in Wei et al. [36], it is more parsimonious to suggest that the A+T-rich region, *trnI* and *trnM* were inverted simultaneously in Braconidae.

Extensive gene rearrangements occur in the two species of Philopteridae. All mitochondrial genes coded on the minority strand are inverted in both species, forming novel patterns, with all genes coded on the majority strand except for *trnQ* in *Campanulotes bidentatus*. Thus, A+T-rich regions are probably inverted independent of the rearrangements of other genes in Philopteridae.

Amongst Aleyrodidae, *Aleurodicus dugesii* and *T. vaporariorum* the tRNAs are rearranged and largely restricted to the tRNA clusters between the A+T-rich region and *nad2*, *nad2* and *cox1*. In the mitochondrial genomes of the other four species, a large segment containing at least *cox3*, *trnG* and *nad3* is remotely inverted.

Based on our analyses of the structures of A+T-rich regions and gene arrangement patterns, inversion of the A+T-rich region is the only character state unique to these 10 species. In both Braconidae and Aleyrodidae, some species show conserved mitochondrial gene arrangement whereas others are extensively rearranged. Thus, the inversion of the A+T-rich region may have occurred prior to extensive gene rearrangements in, at least, some species.

In *C. vestalis*, *Bothriometopus macrocnemis* and *C. bidentatus*, protein-coding genes are extensively inverted. Since replication and transcription processes have been widely considered to bias the occurrence of mutations between two complementary DNA strands [9], [17], the inversion of large number of protein-coding genes should have counteracted some of the affect of reversed replication origin on strand asymmetry.

In eukaryotes, many non-coding regions can be used for the comparison of strand asymmetry between transcribed and untranscribed regions [29], [30]; however, most animal mitochondrial genomes are highly economized with few non-coding regions to facilitate the study of transcription-associated strand asymmetry [44]. Fortunately, in the putative ancestral insect mitochondrial genome, four of the 13 protein-coding genes are located on the minority strand and the total length of protein-coding genes coded on the minority strand accounts for about 2/3 of those of the entire mitochondrial genome. This provided us with the opportunity to compare the strand asymmetry of genes coded on different strands. Contrastingly, in mammal mitochondrial genomes, which are frequently used models for studies of strand asymmetry, only one protein-coding gene is coded on the minority strand, and this gene has been excluded from many [23], [45] but not all [25] studies. Here, we analyzed strand asymmetry for individual protein-coding genes in 120 insect mitochondrial genomes with special reference to the relationship between strand asymmetry and gene direction/genome replication orientation.

### Strand asymmetry on single protein-coding genes

**Figure 4:**
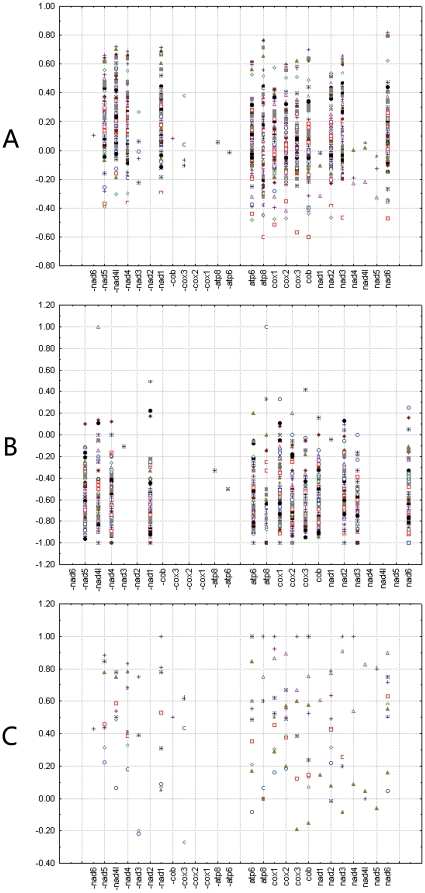
Scatterplots of skews values calculated for third codon positions of individual protein-coding genes. (A) Relationship of AT skew on third codon position of individual gene and gene direction in 120 insect species. (B) Relationship of GC skew on individual gene and gene direction in 110 insect mitochondrial genomes with normal replication origin. (C) Relationship of GC skew on individual gene and gene direction in 10 insect mitochondrial genomes with inverted replication origin. Gene name with minus indicates that the gene is encoded on minority strand, while without minus indicates on majority strand.

Third codon positions are less affected by selection on amino acids, but many third codon positions are not free of change because of the existence of two-fold redundant codon positions. Four-fold third codon positions, which can freely alternate between all nucleotides without changing the resulting amino acid, are considered to have little or no affect on selection. Here, we calculated AT and GC skews for all codon positions, third codon positions, two-fold redundant third codon positions, and four-fold redundant third codon positions for individual protein-coding genes (Figure 4, Figure S1 and Table S1).

At all codon positions and two-fold redundant third codon positions, most genes coded on the minority strand show positive AT skew values, whereas most genes coded on the majority strand show negative AT skew values. This is the case in all analyzed insects with normal replication origins and inverted replication origins, except for six Aleyrodidae species with inverted replication origins. In these, some genes coded on the minority strand showed negative AT skew values. Other exceptions are mostly restricted to specific taxa or genes. At all codon positions, genes which coded on the majority strand in all Japygoidea (Diplura), Isoptera, Acrididae (Orthoptera), Archostemata (Coleoptera), Elateriformia (Coleoptera), and Archaeognatha showed positive AT skew values. All genes coded on the majority strand of all Oedipodinae have negative AT skew values. At two-fold redundant third codon positions, *nad4L*, coded on the minority strand, showed negative AT skew values in most species unlike other genes coded on the minority strand. At third codon positions and four-fold redundant third codon positions, most genes coded on the minority strand showed positive AT skew values, however, genes coded on the majority strand showed either positive or negative AT skew values (Figure 4A and Figure S1).

Most genes coded on both the majority and minority strands have negative GC skew values in genomes with normal replication origin (Figure 4B and Figure S1); whereas most genes coded on both the majority and the minority strands show positive GC skew values in the genomes of the 10 species with reversed strand asymmetry over the entire majority strand (Figure 4C and Figure S1).

We tested the correlation between the sign of skew values on individual genes and gene direction/genome replication orientation using contingency table and chi-square test. The results support the idea that the sign of AT skew on individual genes is associated with gene direction, but the sign of GC skew on individual genes is not associated with gene direction at third codon positions and four-fold redundant third codon positions. The *p*-values support the correlation between the sign of GC skews of individual gene and gene direction at all codon positions and two-fold redundant third codon positions, however, the chi-square values were lower than that for AT skew. The sign of both AT and GC skew values are associated with replication origin, however, the chi-square values were lower for AT skew than for GC skew (Table 1). In conclusion, the sign of GC skew is associated with replication orientation but not associated with gene direction. The sign of AT skew varies with gene direction, replication and codon position.

**Table 1:** Contingency table and chi-square test for gene direction/replication orientation and sign of skew value.

Factors	All codon positions		Third codon position		R2P3		R4P3	
	*X^2^*	*P*	*X^2^*	*P*	*X^2^*	*P*	*X^2^*	*P*
RO×AT	37.8846	0.0001	170.8983	0.0001	33.5929	0.0001	332.2560	0.0001
RO×GC	1032.0096	0.0001	1046.7640	0.0001	459.8712	0.0001	351.8618	0.0001
GD×AT	784.6107	0.0001	130.7445	0.0001	263.3359	0.0001	5.2118	0.0224
GD×GC	5.6456	0.0175	0.0676	0.7948	102.6981	0.0001	0.1481	0.7004

All mitochondrial genomes used in this study were included in analysis. R2P3, two-fold redundant third codon positions; R4P3, four-fold redundant third codon positions; *P*, significant value; *X^2^*, Chi-square value; RO×AT, replication orientation and sign of AT skew; RO×GC, replication orientation and sign of GC skew; GD×AT, gene direction and sign of AT skew; GD×GC, gene direction and sign of GC skew.

## Discussion

### Reversal of strand asymmetry in insect mitochondrial genomes

We conducted a broad survey of strand asymmetry in 120 insects and showed that reversal of GC skew sign over the entire majority strand evolved three times in insects. Further we demonstrated that reversal of GC skew sign over the entire majority strand appears to be correlated phylogeneticly. This is in contrast with other animal taxa, in which reversal of strand asymmetry is randomly distributed [12], [18], though fewer species across a larger phylogenetic space have been sampled. More mitochondrial genomes sequences are needed to confirm the correlation between the reversal of strand asymmetry in mitochondrial genomes and taxonomic groups. That reversal of strand asymmetry was caused by inversion of replication origin was demonstrated by the examination of replication-related elements in the A+T-rich region [12], [18]. All species with reversal of strand asymmetry over the entire mitochondrial genome were found to have accelerated gene rearrangement rates, whereas species with accelerated mitochondrial genomes rearrangement did not always show reversal of strand asymmetry. This may indicate that inversion of the A+T-rich region, leading to reversal of strand asymmetry, is a type of gene rearrangement event unique to mitochondrial genomes. The causes of frequent gene rearrangements in mitochondrial genomes have yet to be examined [46].

### Skew values of entire mitochondrial genomes and individual genes

By comparing six protein-coding genes in 49 metazoan mitochondrial genomes, Hassanin et al. (2005) found that absolute values for GC are always higher than those of AT skews at all codon positions and suggested that strand asymmetry is best reflected in the GC skew. Skew values on individual genes for all codon positions could help to explain this phenomenon. GC skews of different genes coded on the same strand are all positive or negative, whereas the AT skews of different genes coded on the same strand are either positive or negative depending on the direction of the gene, thus the GC skew on a strand is the accumulative effect of all genes, and the AT skew on a strand is the homogenized result of those AT skews with different signs on individual genes. Our conclusion is that the criterion for detecting reversal of strand asymmetry on mitochondrial genomes should be the sign of GC skew values not AT skew values.

### Strand asymmetry and genome replication/gene transcription

Strand asymmetry is the consequence of selection and asymmetric patterns of mutation between the two strands [47], [48]. Two processes are widely accepted to bias mutations, i.e., replication and transcription. It is well supported that the deamination of A and C at exposed single-stranded regions results in an increase of C and A content on the complementary sequences [8], [9], [17].

Although some [23] but not all [25] previous analyses excluded genes encoded on the minority strand or genes with short length, and limited to the positions with weakest selection pressure [23], [25], the results on vertebrate mitochondrial genomes show that different types of mutations respond differently to DssH gradient [25], indicating that mutation patterns in mitochondrial DNA were more complicated than previously thought.

Under the widely accepted “strand-displacement model” of animal mitochondrial genome replication [49], [50], [51], [52], the parent majority strand is first used as a template to synthesize the nascent minority strand, i.e., the leading strand, with the parent minority strand left single-stranded, and consequently experienced more A and C deaminations. Thus, the synthesized nascent majority strand, i.e., lagging strand, using parent minority strand as a template, has more A and C than the leading strand. This replication theory is congruent with our observations that more A and C are present on the entire majority strand in most mitochondrial genomes with normal replication origin; whereas there is less A and C on the entire majority strand in most genomes with inverted replication origin. On a protein-coding gene sequence, all positions should be affected by both selection and mutation, except for four-fold third codon positions. In our study, the sign of GC skew values on individual protein-coding genes was almost identical among analysis for four different partitions of gene sequences, indicating that deaminations occurring during replication, in addition to other mutations, contributed more than selection on amino acid sequences to the strand composition of G and C. This is congruent with the hypothesis, for mammalian mitochondrial genomes and nuclear DNA, that one of the crucial processes for the origin of strand asymmetry is the spontaneous deamination of C and A in the H-strand (referred to as the “minority strand” in insects) during replication, with deamination of C being twofold higher than deamination of A [22], [23], [24].

Under the transcription model, while RNA is being synthesized on the transcribed strand of DNA, the nontranscribed DNA strand remains transiently single stranded. It has been shown experimentally that transcription biases the mutational patterns between the transcribed and nontranscribed strands [53]. In mammalian mitochondria, both strands are transcribed as a single polycistronic precursor RNA [54]. Therefore, Hassanin et al. (2005) suggested that transcription can be considered to be an asymmetric process, because the L-strand (named as “majority strand” in insects) is transcribed two or three times more frequently than the H-strand (named as “minority strand” in insects), and the H-strand is expected to be more prone to deamination and transcription-coupled repair mutations due to its single-stranded state during transcription of the L-strand. Thus, regions on the same strand have the same tendency of A and C content variation produced by transcription. However, this model does not explain the variation of AT skew values depending on gene direction examined at all codon positions.

It is likely that there is a second initiation site for H-strand transcription in mammalian mitochondria, one that produces RNA transcript spanning the rDNA region [55]. Furthermore, an alternative model of transcription in *Drosophila* proposed that four major blocks of genes, coded on the same strand, have unique transcription initiation sites upstream of their coding region [56], [57] This implies that transcription of one coding region is possible [8]. We suggest that similar multiple polycistronic transcription models may exist in insect mitochondria, under which each block of genes coded on the same strand are transcribed in at least one transcript, resulting in only antisense strands being transcribed. Thus, antisense strands are paired with nascent mRNA, and sense strands are exposed in a single-stranded state, and more A and C deamination occurs during transcription. This leads to the increase of A and C content on the nascent antisense strand, but hardly affects A and C content on the nascent sense strand. At all codon positions there is more A than T in genes coded on minority strand while more T than A in genes coded on majority strand both with normal and inverted replication origin, indicating that selection on amino acid sequence, in addition to mutation during transcription, is stronger than mutation during replication. At four-fold redundant third codon positions, where selection has little or no effect on nucleotide composition, AT skew values for most genes, coded on both majority and minority strand in mitochondrial genomes with normal replication origin, are positive. Contrastingly, those with inverted replication origin are negative, indicating that replication process has a stronger affect on A and T content than transcription. In a previous study, Faith and Polleck also concluded that transcription plays no detectable role in producing the gradients of asymmetry at redundant sites by separately analysing the four-fold redundant sites in *nad6*, the only gene transcribed on the minority strand in vertebrate mitochondrial genome [25].

In conclusion, we hope to have demonstrated the relative contributions of selection on amino acid sequences and mutations during replication and transcription to strand asymmetry in animal mitochondrial DNA. Further we show that replication plays a key role in generating strand asymmetry, and that the relative contribution of selection and mutation varies among nucleotides. This, in turn, indicates that multifactorial studies on mutation patterns are necessary to uncover mutation processes in insect mitochondrial genomes.

Our results not only shed significant light on the mechanisms underlying strand asymmetry, but may also contribute to genome-wide studies of replication and transcription mechanisms.

## Materials and Methods

### Genome and gene acquisition

One hundred and twenty insect mitochondrial genomes were used for strand asymmetry analyses, belonging to 20 orders in three classes of insects (Table S1), including all those available in GenBank at the inception of this study and two recently sequenced ones [36]. Sequences of whole mitochondrial genome strands and individual protein-coding genes were downloaded from the Mitome database [58].

### Calculation of skew values

For each mitochondrial genome, AT and GC skews were calculated for the majority strand, all codon positions, third codon positions, two-fold redundant third codon positions and four-fold redundant third codon positions of protein-coding genes. If a gene was coded on the majority strand, the sense strand was used, whereas if the gene was coded on the minority strand, the antisense strand was used for calculation.

Statistical analyses were conducted in DPS version 9.50 [59]. Contingency table and Chi-square tests for categorical data were used to estimate the association between gene direction and the sign of AT and GC skews, as well as between the replication orientation and the sign of AT and GC skews.

## Supporting Information

Table S1 Mitochondrial genomes used in strand asymmetry analyses and skew values calculated for the whole mitochondrial genomes and individual protein-coding genes. All-AT, AT skew values for all codon positions of individual genes; All-GC, GC skew values for all codon positions of individual genes; P3-AT, AT skew values for third codon positions of individual genes; P3-GC, GC skew values for third codon positions of individual genes; R2P3-AT, AT skew values for two-fold redundant third codon positions of individual genes; R2P3-GC, GC skew values for two-fold redundant third codon positions of individual genes; R4P3-AT, AT skew values for four-fold redundant third codon positions of individual genes; R2P3-GC, GC skew values for four-fold redundant third codon positions of individual genes.
(2.82 MB XLS)

Figure S1 Scatterplots of AT and GC skews values calculated for all codon positions, two-fold redundant third codon positions and four-fold redundant third codon positions of individual protein-coding genes in insect mitochondrial genomes. A. Scatterplots of AT skews values calculated for all codon positions of individual protein-coding genes in 120 insect mitochondrial genomes. B. Scatterplots of AT skews values calculated for two-fold redundant third codon positions of individual protein-coding genes in 120 insect mitochondrial genomes. C. Scatterplots of AT skews values calculated for four-fold redundant third codon positions of individual protein-coding genes in 120 insect mitochondrial genomes. D. Scatterplots of GC skews values calculated for all codon positions of individual protein-coding genes in 10 insect mitochondrial genomes with inverted replication origin. E. Scatterplots of GC skews values calculated for all codon positions of individual protein-coding genes in 110 insect mitochondrial genomes with normal replication origin. F. Scatterplots of GC skews values calculated for two-fold redundant third codon positions of individual protein-coding genes in 10 insect mitochondrial genomes with inverted replication origin. G. Scatterplots of GC skews values calculated for two-fold redundant third codon positions of individual protein-coding genes in 110 insect mitochondrial genomes with normal replication origin. H. Scatterplots of GC skews values calculated for four-fold redundant third codon positions of individual protein-coding genes in 10 insect mitochondrial genomes with inverted replication origin. I. Scatterplots of GC skews values calculated for four-fold redundant third codon positions of individual protein-coding genes in 110 insect mitochondrial genomes with normal replication origin. Gene name with minus indicates that the gene is coded on minority strand, while without minus indicates on majority strand.
(0.06 MB PDF)

## References

[1] Boore JL (1999). Animal mitochondrial genomes.. Nucleic Acids Research.

[2] Zhang DX, Hewitt GM (1997). Insect mitochondrial control region: a review of its structure, evolution and usefulness in evolutionary studies.. Biochemical Systematics and Ecology.

[3] Ballard JWO, Whitlock MC (2004). The incomplete natural history of mitochondria.. Molecular Ecology.

[4] Nikolaou C, Almirantis Y (2006). Deviations from Chargaff's second parity rule in organellar DNA - insights into the evolution of organellar genomes.. Gene.

[5] Albrecht-Buehler G (2006). Asymptotically increasing compliance of genomes with Chargaff's second parity rules through inversions and inverted transpositions.. Proceedings of the National Academy of Sciences of the United States of America.

[6] Perna NT, Kocher TD (1995). Patterns of nucleotide composition at fourfold degenerate sites of animal mitochondrial genomes.. Journal of Molecular Evolution.

[7] Simon C, Frati F, Beckenbach A, Crespi B, Liu H (1994). Evolution, weighting, and phylogenetic utility of mitochondrial gene sequences and a compilation of conserved polymerase chain reaction primers.. Annals of the Entomological Society of America.

[8] Cameron SL, Johnson KP, Whiting MF (2007). The mitochondrial genome of the screamer louse *Bothriometopus* (Phthiraptera: Ischnocera): effects of extensive gene rearrangements on the evolution of the genome.. Journal of Molecular Evolution.

[9] Hassanin A, Leger N, Deutsch J (2005). Evidence for multiple reversals of asymmetric mutational constraints during the evolution of the mitochondrial genome of Metazoa, and consequences for phylogenetic inferences.. Systematic Biology.

[10] Kilpert F, Podsiadlowski L (2006). The complete mitochondrial genome of the common sea slater, *Ligia oceanica* (Crustacea, Isopoda) bears a novel gene order and unusual control region features.. BMC Genomics.

[11] Masta SE, Longhorn SJ, Boore JL (2009). Arachnid relationships based on mitochondrial genomes: Asymmetric nucleotide and amino acid bias affects phylogenetic analyses.. Molecular Phylogenetics and Evolution.

[12] Hassanin A (2006). Phylogeny of Arthropoda inferred from mitochondrial sequences: strategies for limiting the misleading effects of multiple changes in pattern and rates of substitution.. Molecular Phylogenetics and Evolution.

[13] Min XJ, Hickey DA (2007). DNA asymmetric strand bias affects the amino acid composition of mitochondrial proteins.. DNA Research.

[14] Helfenbein KG, Brown WM, Boore JL (2001). The complete mitochondrial genome of the articulate brachiopod *Terebratalia transversa*.. Molecular Biology and Evolution.

[15] Scouras A, Smith MJ (2006). The complete mitochondrial genomes of the sea lily *Gymnocrinus richeri* and the feather star *Phanogenia gracilis*: signature nucleotide bias and unique nad4L gene rearrangement within crinoids.. Molecular Phylogenetics and Evolution.

[16] Wang XZ, Wang J, He SP, Mayden RL (2007). The complete mitochondrial genome of the Chinese hook snout carp *Opsariichthys bidens* (Actinopterygii: Cyprinifonnes) and an altemative pattem of mitogenomic evolution in vertebrate.. Gene.

[17] Francino MP, Ochman H (1997). Strand asymmetries in DNA evolution.. Trends in Genetics.

[18] Fonseca MM, Posada D, Harris DJ (2008). Inverted replication of vertebrate mitochondria.. Molecular Biology and Evolution.

[19] Nikolaou C, Almirantis Y (2005). A study on the correlation of nucleotide skews and the positioning of the origin of replication: different modes of replication in bacterial species.. Nucleic Acids Research.

[20] Sancar A, Sancar GB (1988). DNA repair enzymes.. Annual Reviews in Biochemistry.

[21] Frederico LA, Kunkel TA, Shaw BR (1990). A sensitive genetic assay for the detection of cytosine deamination: determination of rate constants and the activation energy.. Biochemistry.

[22] Lindahl T (1993). Instability and decay of the primary structure of DNA.. Nature.

[23] Reyes A, Gissi C, Pesole G, Saccone C (1998). Asymmetrical directional mutation pressure in the mitochondrial genome of mammals.. Molecular Biology and Evolution.

[24] Tanaka M, Ozawa T (1994). Strand asymmetry in human mitochondrial DNA mutations.. Genomics.

[25] Faith JJ, Pollock DD (2003). Likelihood analysis of asymmetrical mutation bias gradients in vertebrate mitochondrial genomes.. Genetics.

[26] Francino MP, Chao L, Riley MA, Ochman H (1996). Asymmetries generated by transcription-coupled repair in enterobacterial genes.. Science.

[27] Beletskii A, Bhagwat AS (1998). Correlation between transcription and C to T mutations in the non-transcribed DNA strand.. Biological Chemistry Hoppe-Seyler.

[28] Francino MP, Ochman H (2001). Deamination as the basis of strand-asymmetric evolution in transcribed *Escherichia coli* sequences.. Molecular Biology and Evolution.

[29] Green P, Ewing B, Miller W, Thomas PJ, Green ED (2003). Transcription-associated mutational asymmetry in mammalian evolution.. Nature Genetics.

[30] Touchon M, Arneodo A, d'Aubenton-Carafa Y, Thermes C (2004). Transcription-coupled and splicing-coupled strand asymmetries in eukaryotic genomes.. Nucleic acids research.

[31] Haag-Liautard C, Coffey N, Houle D, Lynch M, Charlesworth B (2008). Direct estimation of the mitochondrial DNA mutation rate in *Drosophila melanogaster*.. PLoS Biology.

[32] Stewart JB, Freyer C, Elson JL, Wredenberg A, Cansu Z (2008). Strong purifying selection in transmission of mammalian mitochondrial DNA.. PLoS Biology.

[33] Denver DR, Morris K, Lynch M, Vassilieva LL, Thomas WK (2000). High direct estimate of the mutation rate in the mitochondrial genome of *Caenorhabditis elegans*.. Science.

[34] Montooth KL, Rand DM (2008). The spectrum of mitochondrial mutation differs across species.. PLoS Biology.

[35] Rocha EPC, Touchon M, Feil EJ (2006). Similar compositional biases are caused by very different mutational effects.. Genome Research.

[36] Wei SJ, Min S, Sharkey MJ, Achterberg C, Chen XX (2010). Comparative mitogenomics of Braconidae (Insecta: Hymenoptera) and the phylogenetic utility of mitochondrial genomes with special reference to Holometabolous insects.. BMC Genomics.

[37] Cha SY, Yoon HJ, Lee EM, Yoon MH, Hwang JS (2007). The complete nucleotide sequence and gene organization of the mitochondrial genome of the bumblebee, *Bombus ignitus* (Hymenoptera: Apidae).. Gene.

[38] Thao M, Baumann L, Baumann P (2004). Organization of the mitochondrial genomes of whiteflies, aphids, and psyllids (Hemiptera, Sternorrhyncha).. BMC Evolutionary Biology.

[39] Covacin C, Shao R, Cameron S, Barker SC (2006). Extraordinary number of gene rearrangements in the mitochondrial genomes of lice (Phthiraptera: Insecta).. Insect Molecular Biology.

[40] Wei SJ, Shi M, He JH, Sharkey MJ, Chen XX (2009). The complete mitochondrial genome of *Diadegma semiclausum* (Hymenoptera: Ichneumonidae) indicates extensive independent evolutionary events.. Genome.

[41] Oliveira D, Raychoudhury R, Lavrov DV, Werren JH (2008). Rapidly evolving mitochondrial genome and directional selection in mitochondrial genes in the parasitic wasp *Nasonia* (Hymenoptera: Pteromalidae).. Molecular Biology and Evolution.

[42] Shao RF, Barker SC (2003). The highly rearranged mitochondrial genome of the plague thrips, *Thrips imaginis* (Insecta: Thysanoptera): convergence of two novel gene boundaries and an extraordinary arrangement of rRNA genes.. Molecular Biology and Evolution.

[43] Dowton M, Castro LR, Campbell SL, Bargon SD, Austin AD (2003). Frequent mitochondrial gene rearrangements at the hymenopteran *nad3*-*nad5* junction.. Journal of Molecular Evolution.

[44] Jermiin LS, Graur D, Crozier RH (1995). Evidence from analyses of intergenic regions for strand-specific directional mutation pressure in metazoan mitochondrial DNA.. Molecular Biology and Evolution.

[45] Raina SZ, Faith JJ, Disotell TR, Seligmann H, Stewart CB (2005). Evolution of base-substitution gradients in primate mitochondrial genomes.. Genome Research.

[46] Dowton M, Castro LR, Austin AD (2002). Mitochondrial gene rearrangements as phylogenetic characters in the invertebrates: the examination of genome ‘morphology’.. Invertebrate Systematics.

[47] Lobry JR (1995). Properties of a general model of DNA evolution under no-strand-bias conditions.. Journal of molecular evolution.

[48] Morton RA, Morton BR (2007). Separating the effects of mutation and selection in producing DNA skew in bacterial chromosomes.. BMC Genomics.

[49] Bogenhagen DF, Clayton DA (2003). The mitochondrial DNA replication bubble has not burst.. Trends in Biochemical Sciences.

[50] Clayton DA (1982). Replication of animal mitochondrial DNA.. Cell.

[51] Miyako K, Irie T, Muta T, Umeda S, Kai Y (1999). 1-methyl-4-phenylpyridinium ion (MPP+) selectively inhibits the replication of mitochondrial DNA.. European Journal of Biochemistry.

[52] Brown TA, Cecconi C, Tkachuk AN, Bustamante C, Clayton DA (2005). Replication of mitochondrial DNA occurs by strand displacement with alternative light-strand origins, not via a strand-coupled mechanism.. Genes & Development.

[53] Beletskii A, Bhagwat AS (1996). Transcription-induced mutations: increase in C to T mutations in the nontranscribed strand during transcription in *Escherichia coli*.. Proceedings of the National Academy of Sciences of the United States of America.

[54] Taanman JW (1999). The mitochondrial genome: structure, transcription, translation and replication.. Biochimica Et Biophysica Acta-Bioenergetics.

[55] Gaspari M, Larsson NG, Gustafsson CM (2004). The transcription machinery in mammalian mitochondria.. Biochimica Et Biophysica Acta-Bioenergetics.

[56] Roberti M, Polosa PL, Bruni F, Musicco C, Gadaleta MN (2003). DmTTF, a novel mitochondrial transcription termination factor that recognises two sequences of *Drosophila melanogaster* mitochondrial DNA.. Nucleic Acids Research.

[57] Roberti M, Bruni F, Polosa PL, Gadaleta MN, Cantatore P (2006). The *Drosophila* termination factor DmTTF regulates in vivo mitochondrial transcription.. Nucleic Acids Research.

[58] Lee YS, Oh J, Kim YU, Kim N, Yang S (2008). Mitome: dynamic and interactive database for comparative mitochondrial genomics in metazoan animals.. Nucleic Acids Research.

[59] Tang QY, Feng MG (2006). DPS Data Processing System—Experimental design, statistical analysis and data mining..

