# Supplementary figures and images for: New Views on Strand Asymmetry in Insect Mitochondrial Genomes
Source: PLoS One. 2010 Sep 15;5(9):e12708. doi: 10.1371/journal.pone.0012708 (PMC2939890; doi:10.1371/journal.pone.0012708)

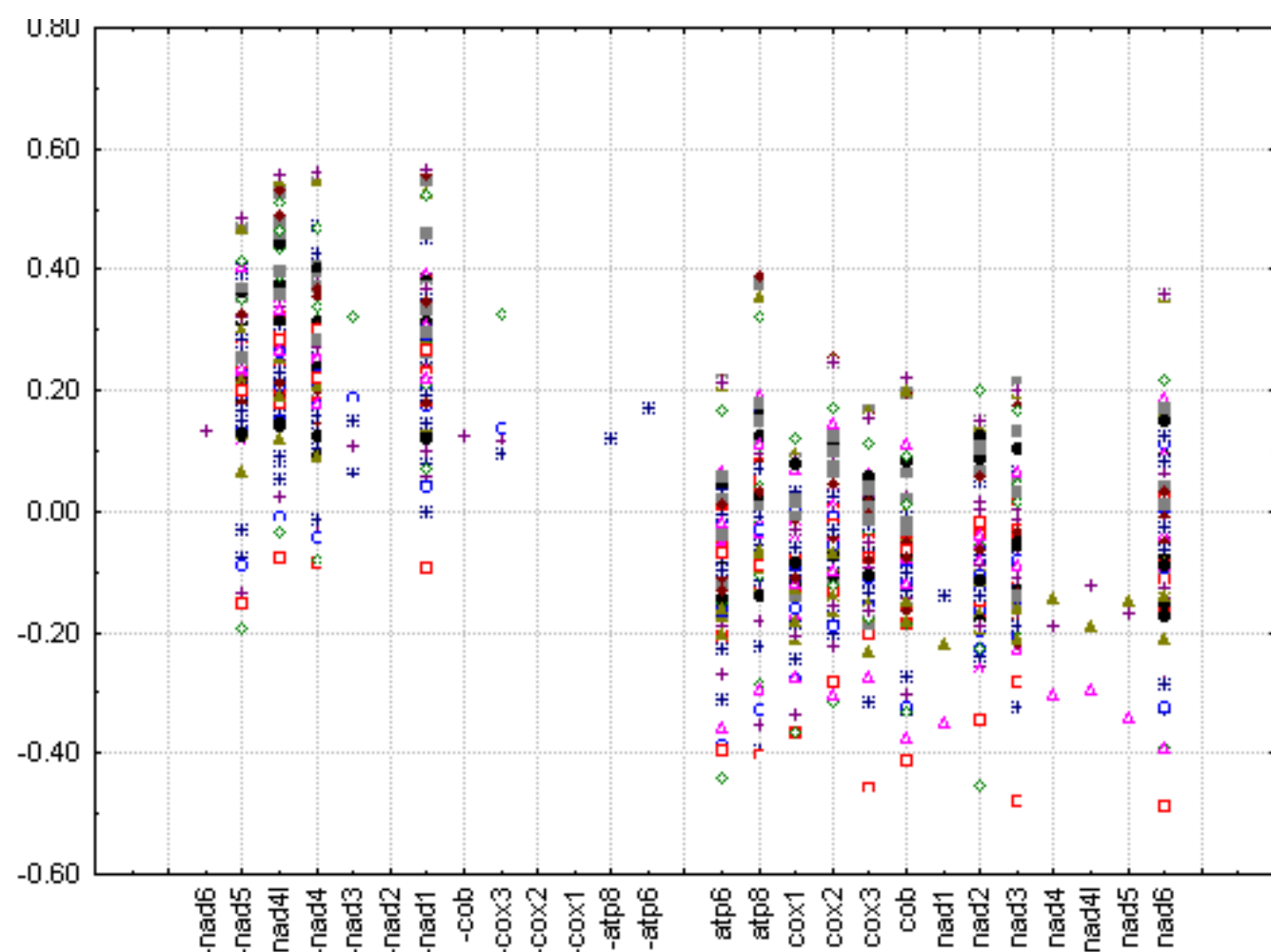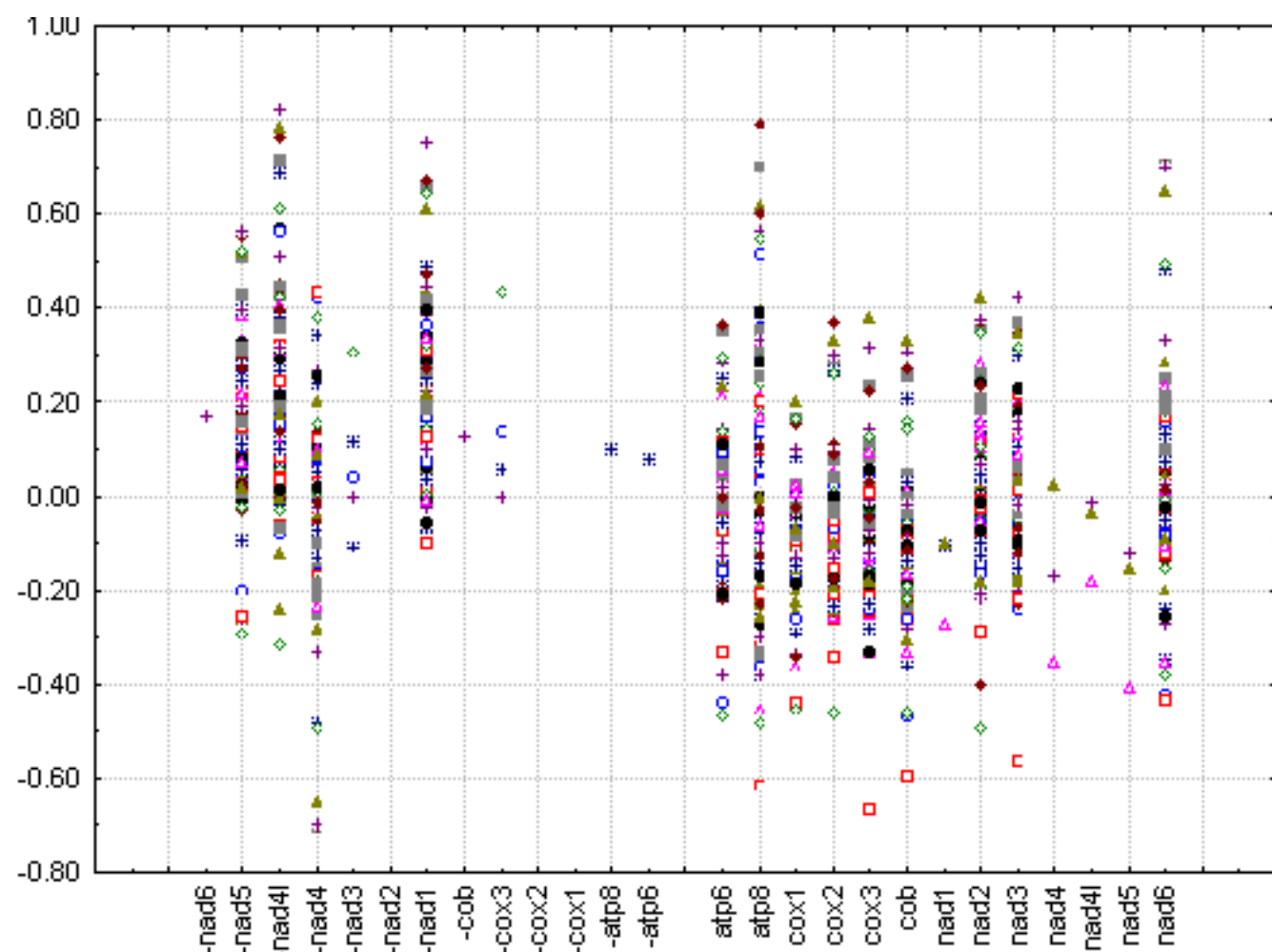

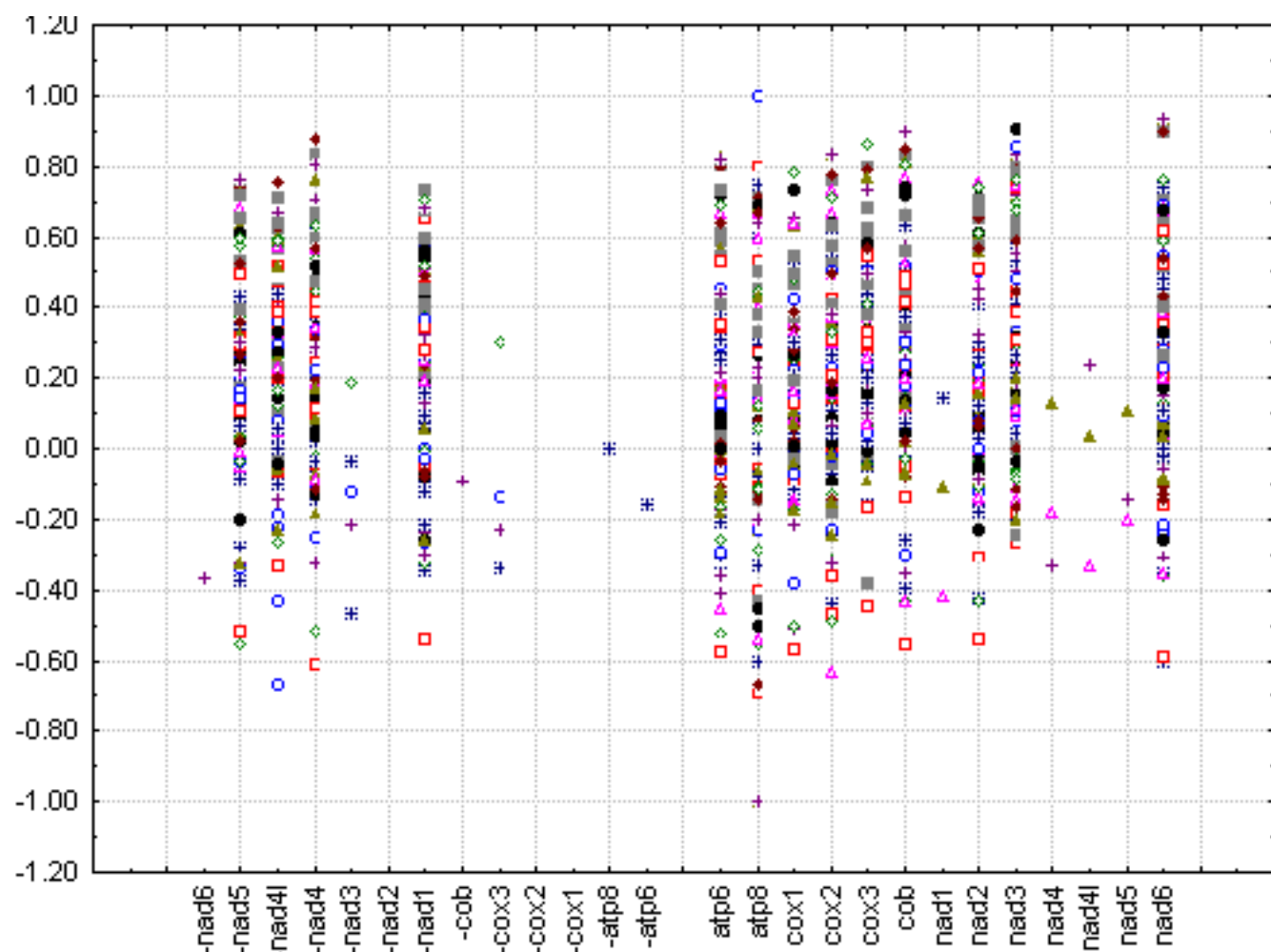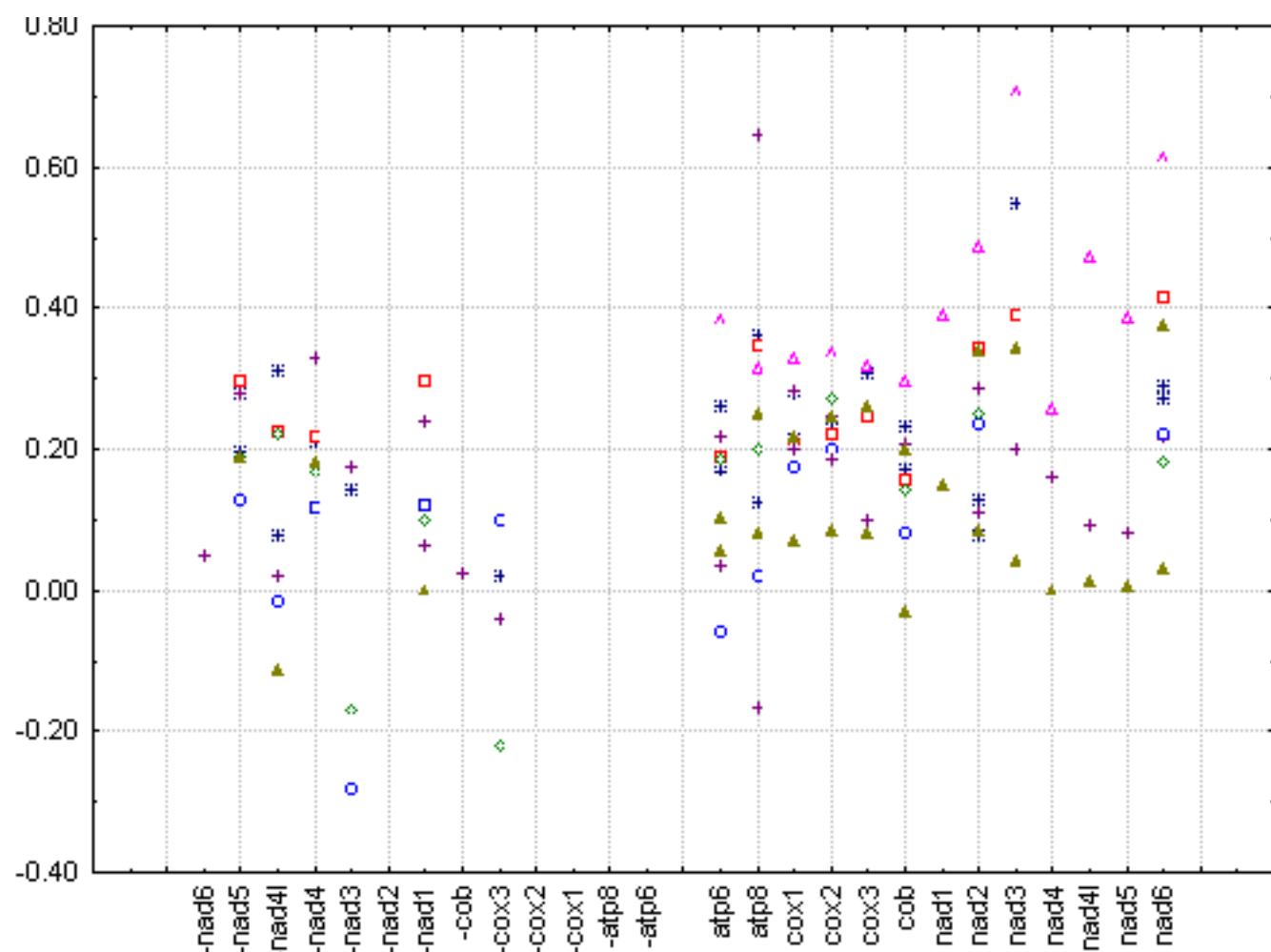

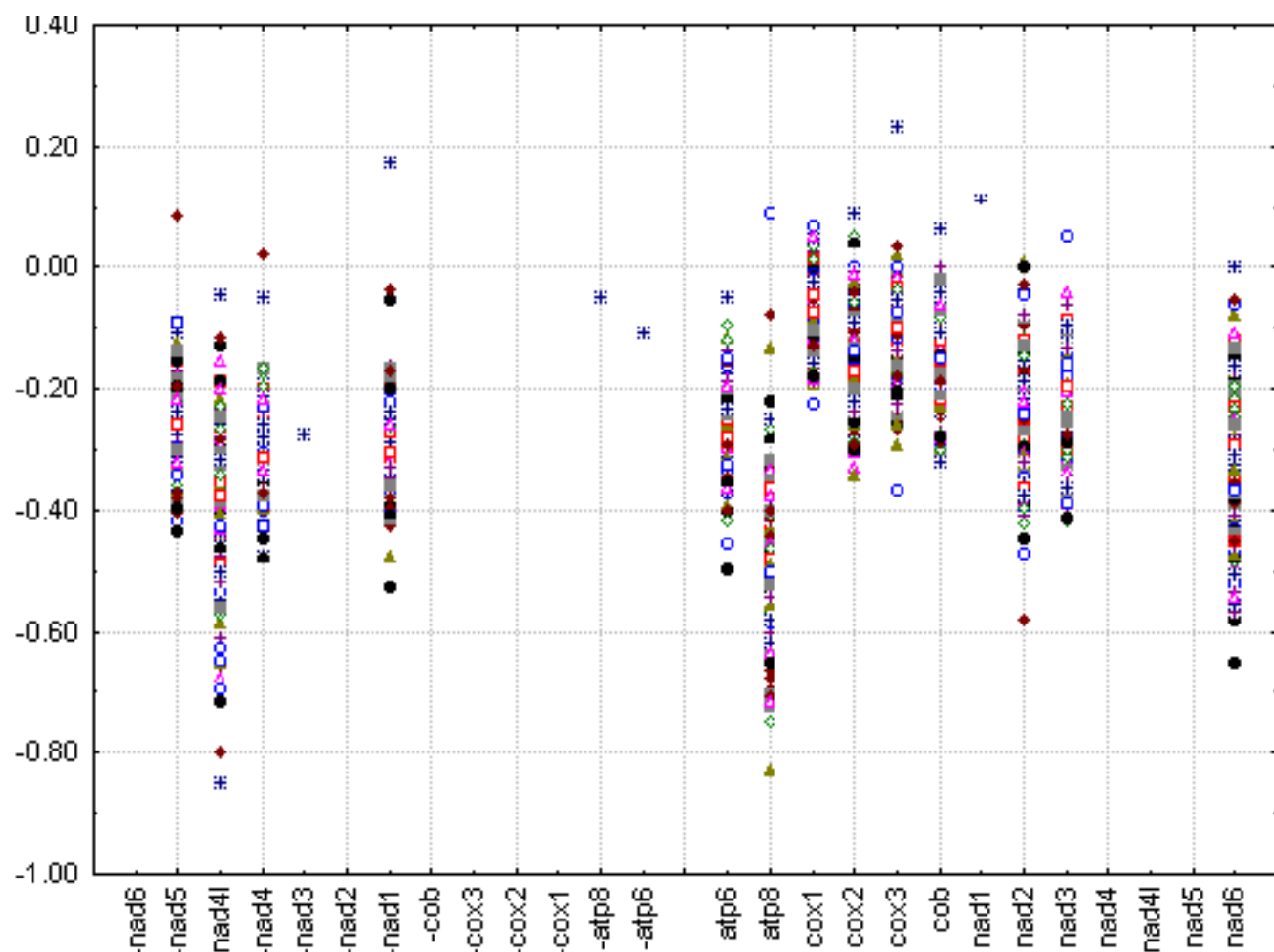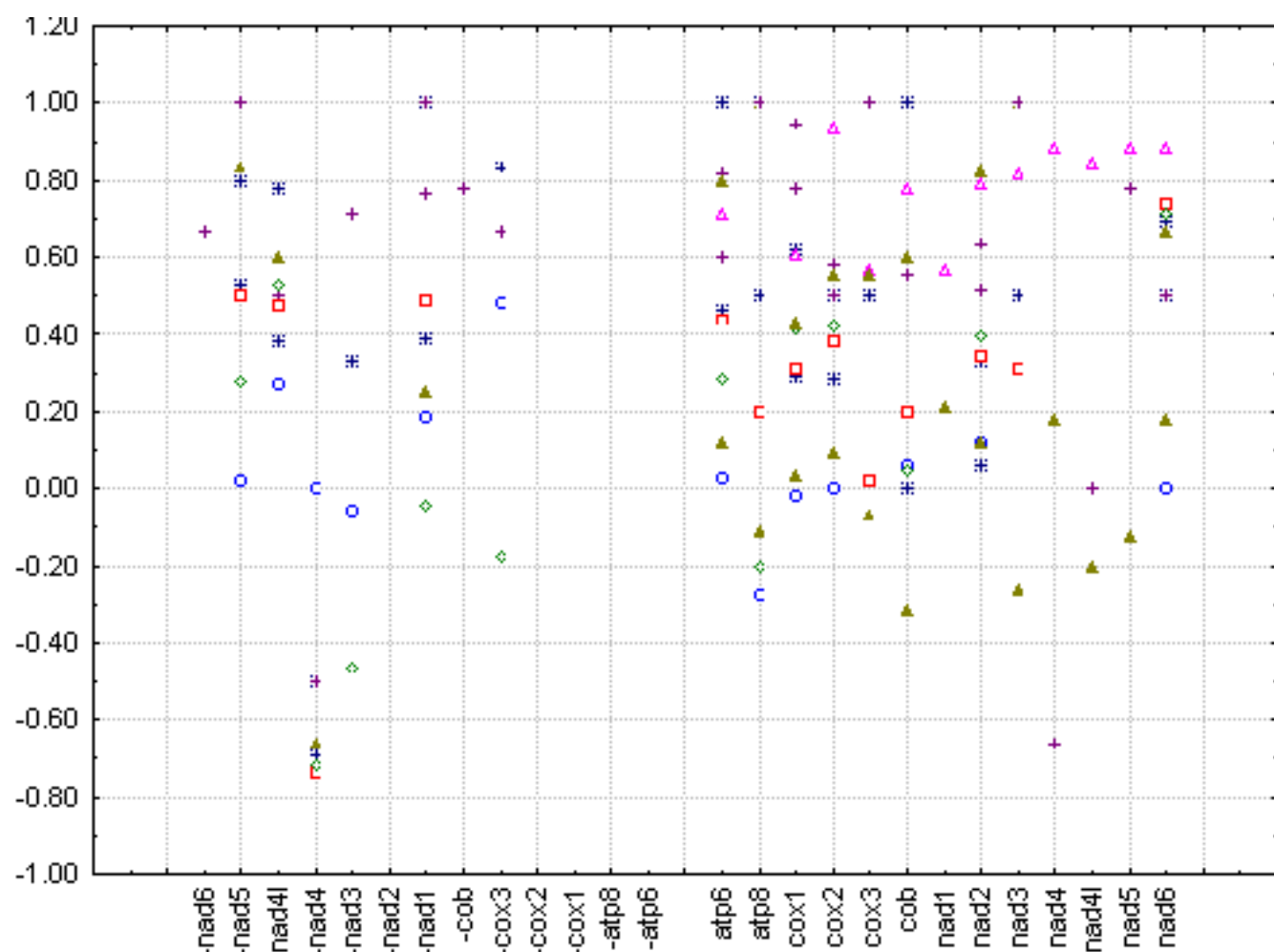

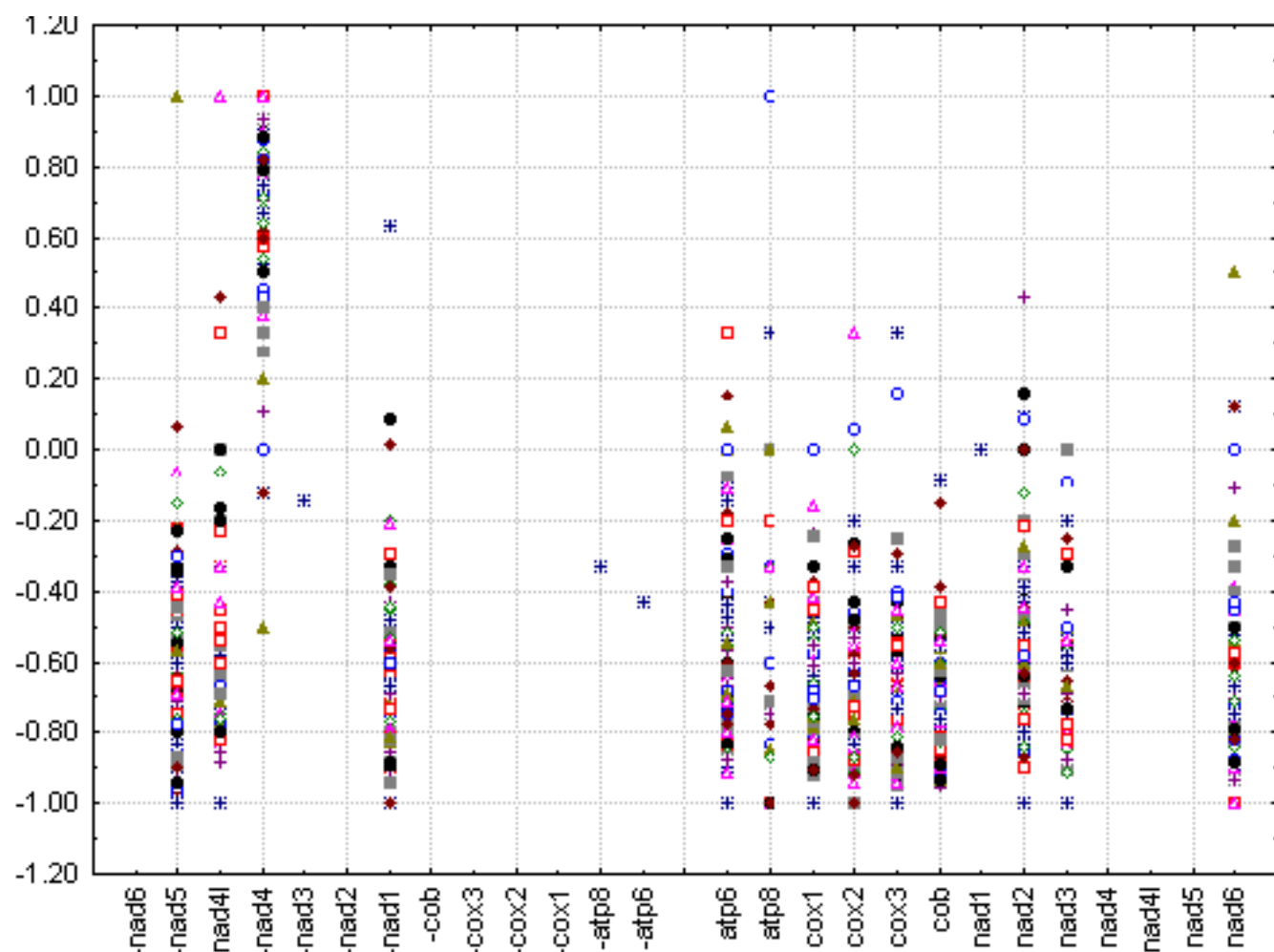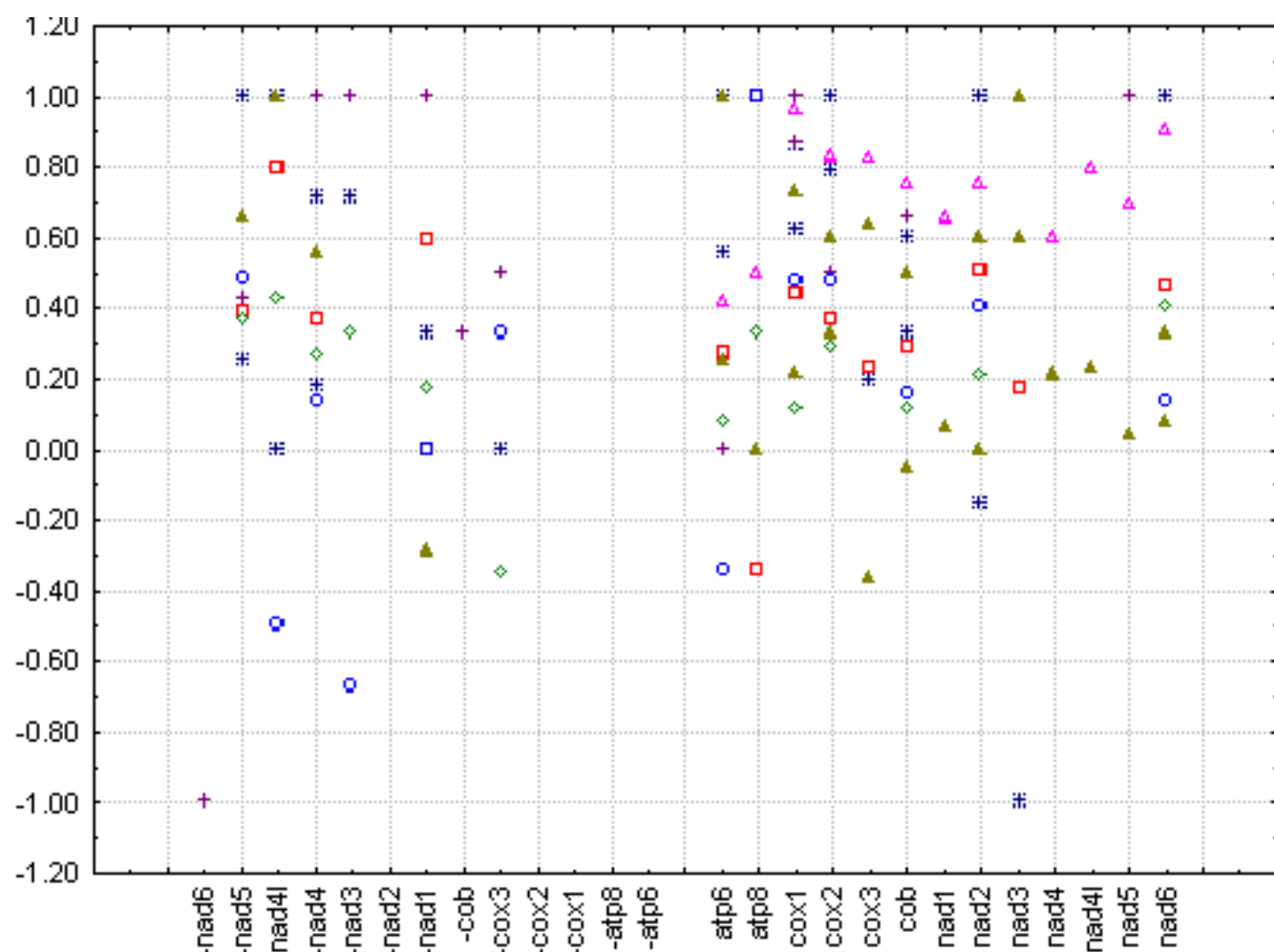

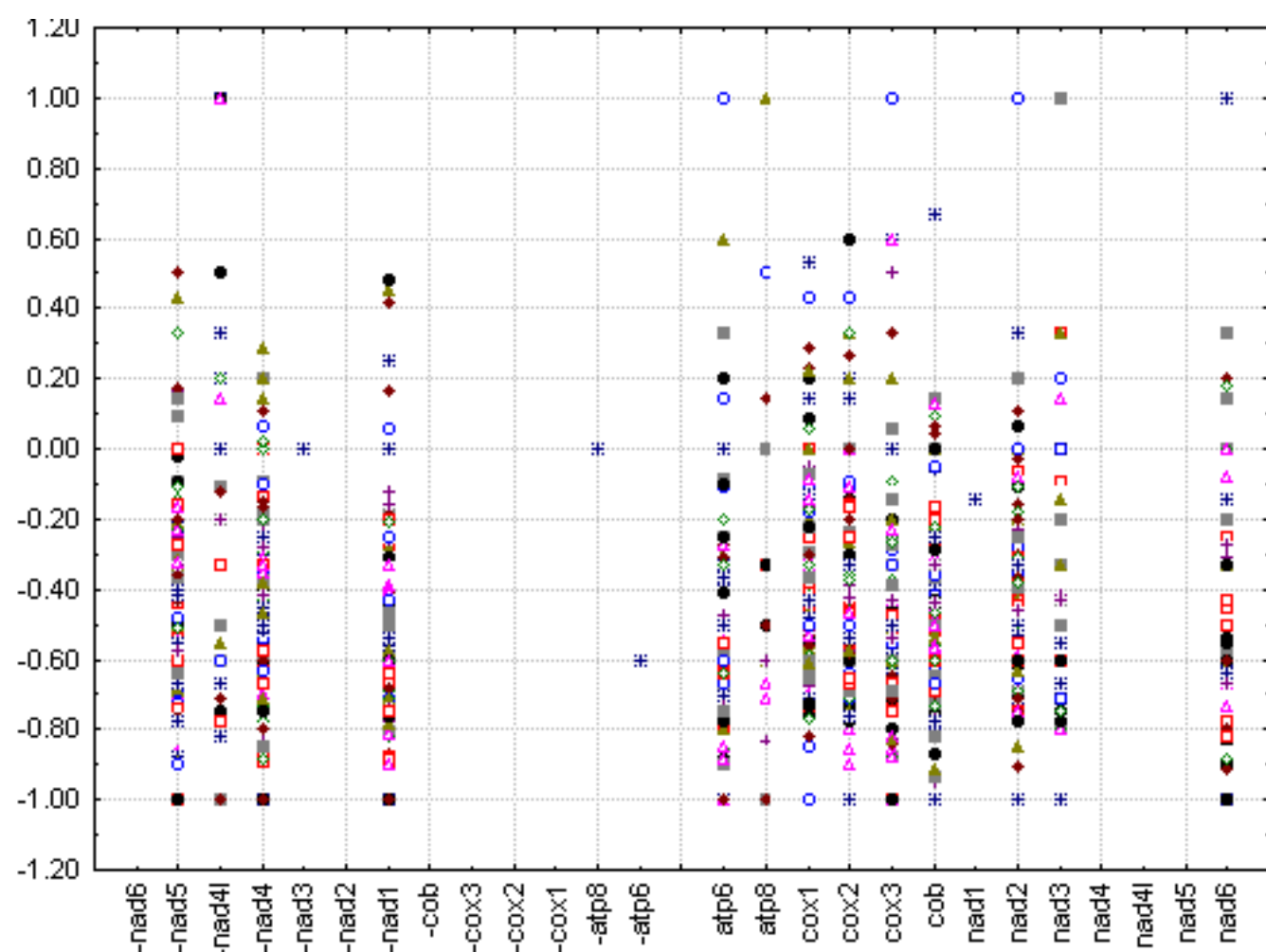

Supplement: Figure S1 — Scatterplots of AT and GC skews values calculated for all codon positions, two-fold redundant third codon positions and four-fold redundant third codon positions of individual protein-coding genes in insect mitochondrial genomes. A. Scatterplots of AT skews values calculated for all codon positions of individual protein-coding genes in 120 insect mitochondrial genomes. B. Scatterplots of AT skews values calculated for two-fold redundant third codon positions of individual protein-coding genes in 120 insect mitochondrial genomes. C. Scatterplots of AT skews values calculated for four-fold redundant third codon positions of individual protein-coding genes in 120 insect mitochondrial genomes. D. Scatterplots of GC skews values calculated for all codon positions of individual protein-coding genes in 10 insect mitochondrial genomes with inverted replication origin. E. Scatterplots of GC skews values calculated for all codon positions of individual protein-coding genes in 110 insect mitochondrial genomes with normal replication origin. F. Scatterplots of GC skews values calculated for two-fold redundant third codon positions of individual protein-coding genes in 10 insect mitochondrial genomes with inverted replication origin. G. Scatterplots of GC skews values calculated for two-fold redundant third codon positions of individual protein-coding genes in 110 insect mitochondrial genomes with normal replication origin. H. Scatterplots of GC skews values calculated for four-fold redundant third codon positions of individual protein-coding genes in 10 insect mitochondrial genomes with inverted replication origin. I. Scatterplots of GC skews values calculated for four-fold redundant third codon positions of individual protein-coding genes in 110 insect mitochondrial genomes with normal replication origin. Gene name with minus indicates that the gene is coded on minority strand, while without minus indicates on majority strand. (0.06 MB PDF) [file pone.0012708.s002.pdf]
